# The emerging role of retromer in neuroprotection

**DOI:** 10.1016/j.ceb.2017.02.004

**Published:** 2017-08

**Authors:** Kirsty J McMillan, Hendrick C Korswagen, Peter J Cullen

**Affiliations:** 1 School of Biochemistry, Biomedical Sciences Building, University of Bristol, BS8 1TD, UK; 2 Hubrecht Institute, Royal Netherlands Academy of Arts and Sciences and University Medical Center Utrecht, Uppsalalaan 8, 3584 CT Utrecht, The Netherlands

## Abstract

Efficient sorting and transportation of integral membrane proteins, such as ion channels, nutrient transporters, signalling receptors, cell–cell and cell–matrix adhesion molecules is essential for the function of cellular organelles and hence organism development and physiology. Retromer is a master controller of integral membrane protein sorting and transport through one of the major sorting station within eukaryotic cells, the endosomal network. Subtle de-regulation of retromer is an emerging theme in the pathoetiology of Parkinson’s disease. Here we summarise recent advances in defining the neuroprotective role of retromer and how its de-regulation may contribute to Parkinson’s disease by interfering with: lysosomal health and protein degradation, association with accessory proteins including the WASH complex and mitochondrial health.

## Highlights

• In the endosomal network retromer retrieves cargo away from the degradative pathway.
• Retromer dysfunction has been implicated in Parkinson’s disease through different mechanisms.
• These include changes in protein association, protein degradation and mitochondria quality control.

**Current Opinion in Cell Biology** 2017, **47**:72–82

This review comes from a themed issue on **Cell Organelles**

Edited by **Bruno Antonny** and **Catherine Rabouille**

For a complete overview see the Issue and the Editorial

Available online 8th April 2017

**http://dx.doi.org/10.1016/j.ceb.2017.02.004**

0955-0674/© 2017 The Authors. Published by Elsevier Ltd. This is an open access article under the CC BY license (http://creativecommons.org/licenses/by/4.0/).

## Introduction

**Figure 1:**
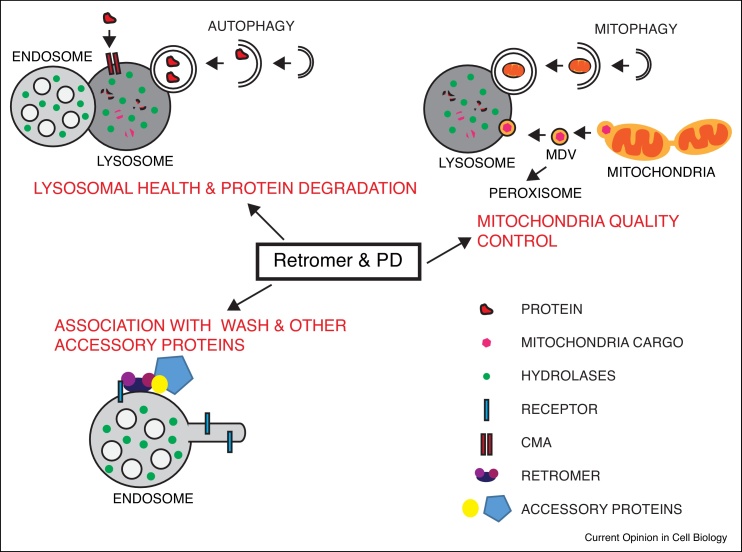
Retromer and Parkinson’s disease. Retromer has been shown to play an important role in the pathology of Parkinson’s disease (PD). The different pathways shown to be affected by retromer dysfunction are illustrated and include changes in lysosomal health and protein degradation, association with accessory proteins including the WASH complex and mitochondrial health. MDV, mitochondria derived vesicles, CMA, chaperone mediated autophagy.

The endosomal network comprises an inter-connected series of intracellular membrane-bound compartments that are present within all eukaryotic cells [1, 2]. This ‘supra-organelle’ serves an essential function in controlling the transport of integral membrane proteins and associated proteins and lipids (together termed ‘cargos’) to a variety of cellular compartments, a function that is vital for development as well as cellular and organismal level physiology [3] (Figure 1).

**Figure 2:**
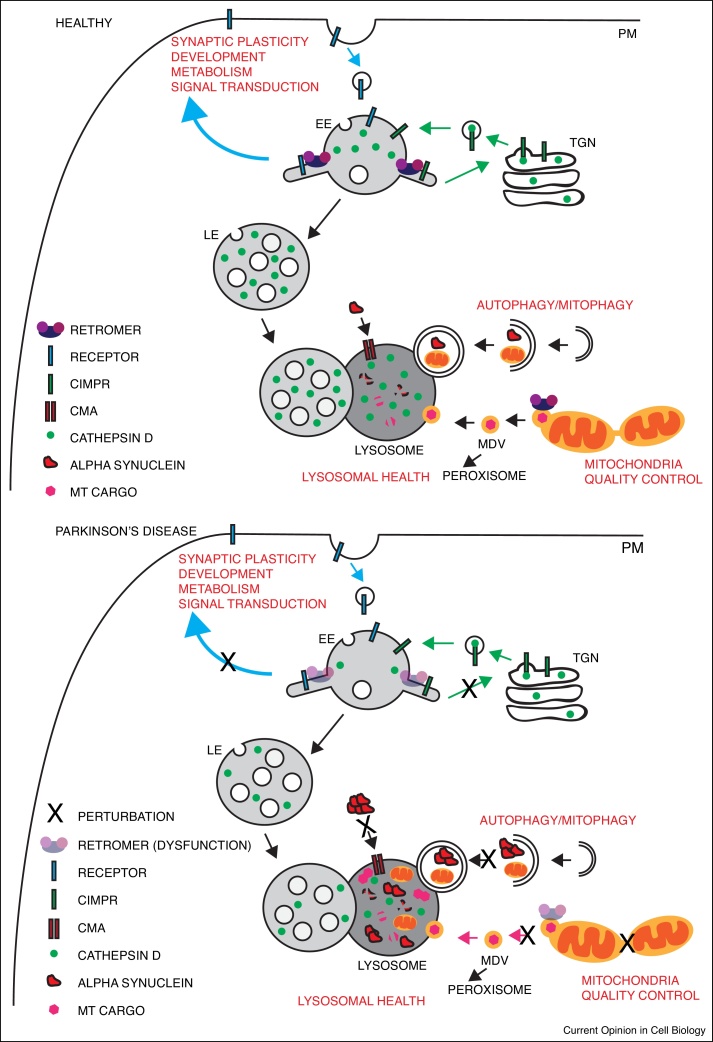
Overview of the endosomal pathways implicated in Parkinson’s disease. In physiological conditions cargo proteins enter the endosomal network where they are either retrieved and recycled (to the plasma membrane (PM), *trans*-golgi network (TGN) or to specialised organelles) or sorted for degradation within the lysosome. Retromer is involved in the retrieval and recycling of cargo away from the degradative pathway. Retromer dysfunction has been implicated in Parkinson’s disease (PD). Several mechanisms are proposed including perturbations in lysosomal health, autophagy flux, mitochondria quality control and the cell surface proteome. Impairment of retromer affects sorting of integral cell surface proteins, which can affect many aspects of plasma membrane function. Furthermore, retromer dysfunction affects trafficking of hydrolases from the TGN to the lysosome (via CI-MPR) affecting the health of the lysosome and resulting in reduced protein degradation. Retromer is also involved in the trafficking of proteins needed in autophagasome formation, chaperone mediated autophagy (CMA) and mitochondria derived vesicles (MDV) all of which are involved in maintaining proteostasis. Impairment of these different pathways are thought to lead to increases in alpha synuclein, the key protein present in Lewy bodies in Parkinson’s disease, as well as other proteins and damaged organelles potentially leading to cell death. In addition, retromer dysfunction has been shown to affect mitochondrial health due to changes in the expression of fission and fusion proteins which may also play a role in the pathology of Parkinson’s disease. EE, early endosome; LE, late endosome, MT, mitochondria.

Cargo can enter the network from the plasma membrane, the biosynthetic pathway and various other routes that include the autophagic pathway [1, 2]. Once within the network cargos have essentially two fates: they are either sorted towards the lysosome for degradation or are retrieved from this fate for recycling. Cargo can be recycled to the cell surface, the biosynthetic pathway (at the level of the *trans*-Golgi network (TGN)) and other organelles that include autophagosomes and lysosome-related organelles (Figure 2) [1, 2]. While we have an advanced understanding of the molecular mechanisms that govern cargo sorting for degradation [4], relatively little is known about the corresponding details of cargo retrieval and recycling [5]. That said, the identification of retromer as one of the evolutionary conserved conductors for orchestrating endosomal cargo retrieval and recycling has begun to shed light on these complex events [2, 6]. In so doing, exciting and new emphasis has been placed on the importance of cargo retrieval and recycling in neuroprotection and age-related neurodegenerative disease [7]. Here we review some of the most recent literature exploring retromer’s role in this context, with a specific focus on Parkinson’s disease (PD), and consider the future direction of this emerging research area.

## Retromer and Parkinson’s disease

Retromer is an ancient and highly conserved heterotrimeric protein complex, consisting of VPS35, VPS29 and VPS26 (two isoforms, A and B, in humans), and its activity is vital for regulating the retrieval and recycling of numerous cargos away from the degradative pathway for delivery to the TGN, the cell surface as well as specialised organelles [2, 6, 8]. Reflecting its central importance, retromer is essential for development [9] and an increasing body of evidence is consistent with retromer serving a neuroprotective role (especially in age-related neuronal health). Furthermore, disruption of retromer has been observed in a number of diseases including PD [7].

PD is the second most common neurodegenerative disorder after Alzheimer’s disease (AD) and affects approximately 1.5% of the population over the age of 65. It is characterised by several motor impairments including tremor, rigidity, akinesia and postural instability, which are known to stem from the progressive loss of dopaminergic neurons within the substantia nigra pars compacta (SNpc) [10, 11]. However, PD is now known to be a multifactorial disorder in that other cell types are affected and symptoms range from depression and cognitive decline to gastrointestinal problems [12]. Understanding the molecular pathways affected in PD is crucial in understanding the pathoetiology of this disease and the exploration of rationale routes for therapeutic intervention.

Retromer was first highlighted in the pathogenesis of PD through the discovery of the VPS35(p.D620N) mutation [8, 9]. While at low frequency, this mutation has been observed in a number of patients with familial as well as sporadic PD and leads to an autosomal dominant late onset form of the disease [13, 14]. Several other rare nonsynonymous mutations have also now been identified within retromer including VPS26A (p.K93E, p.M112V and p.K297X), VPS29 (p.N72H) and several in VPS35 including (p.G51S, p.P316A, p.R524W and p.L774M) (Table 1) [13, 14, 15, 16, 17, 18, 19, 20]. For those mutations that have been analysed assembly of the retromer complex and its endosomal localisation are generally unperturbed, indicating that these mutations do not cause a catastrophic trafficking defect, consistent with the patients having a late onset of disease [13, 14, 15, 16, 21•, 22•].

**Table 1:** Summary of clinical features of patients with retromer mutations

Gene	NT change	AA change	No. of patients	Age	AAO	Initial symptom	Clinical diagnosis
VPS35	c.G1858A	p.D620N	Various	47–54	Average 51.4	RESTING TREMOR	PD
	c.C2320A	p.L774M^a^	2	58/82	51/73	RESTING TREMOR	PD
	c.A1819G	p.M607V	1	80	76	N/A	PD
	c.A1796G	p.H599R	1	72	54	N/A	PD
	c.T1679C	p.I560T^a^	1	75	68	N/A	PD
	c.C1570T	p.R524W	1	46	37	MICROGRAPHIA	PD
	c.C946T	p.P316S^a^	2	N/A	52/54	N/A	PD
	c.T723G	p.I241M	1	74	72	POSTURAL TREMOR	PD
	c.G171A	p.M57I	1	75	62	RESTING TREMOR	PD
	c.G151S	p.G51S^a^	2	61/76	57/64	TREMOR	DPD/PD
	c.A96T	p.R32S	1	N/A	N/A	N/A	PD

VPS26A	c.A889T	p.K297X	1	90	70	BRADYKINESIA	DPD
	c.A334G	p.M112V	1	73	51	FREEZING GAIT	PSP
	c.A277G	p.K93E	2	72/76	58/56	POSTURAL INSTABILITY/N/A	MSA/PD
	c.G380A	p.R127H^a^	1	78	70	BRADYKINESIA	DPD
	c.a922g	p.N308D^a^	1	77	73	RIGIDITY	PSP

VPS29	c.A216C	P.N72H^a^	1	82	70	NA	PD

NT, nucleotide; AA, amino acid; No, number; AAO, age at onset; N/A, not applicable; PD, Parkinson’s disease; DPD, dementia with Parkinson’s disease; PSP, progressive supranuclear palsy; MSA, multiple system atrophy.

a Either found in a control or unaffected family member [13, 14, 15, 16, 17, 18, 19, 20].

Whilst the pathogenicity of these new mutations is as of yet unclear they all point towards retromer, and more broadly the process of endosomal cargo retrieval and recycling, as having an important role in the development of PD. Consistent with this, retromer has been shown to be neuroprotective against the pathology of PD. Overexpression of retromer has been shown to rescue against LRRK2 mutant phenotypes *in vitro* and in *Drosophila* models [23, 24]. In addition, VPS35 is protective against the toxin 1-methyl-4-phenyl-1,2,3,6-tetrahydropyridine (MPTP) as well as the pesticide rotenone both of which, induce Parkinsonian phenotypes [24, 25]. VPS35 heterozygote mice (VPS35+/−) exhibit dopaminergic neuron loss and an increase in alpha synuclein expression, whilst mice expressing a deletion of VPS35 specifically in their dopaminergic neurons have a stronger phenotype displaying an enhanced reduction of dopaminergic neurons, increased alpha synuclein expression and motor deficits, all hallmarks of PD [25]. Additionally, VPS35 mRNA levels are decreased in the SNpc of patients with PD further emphasising an important role for retromer in the pathogenesis of not only familial forms of the disease but also sporadic PD [23]. However, overexpression of VPS35 has also been shown to be toxic in primary cortical neurons and in a rat model, suggesting that the level of retromer expression is important and is likely to be highly controlled and regulated [26].

The precise mechanism(s) by which retromer may be involved in the pathology of PD remains unclear. PD has been shown to cause defects in various molecular pathways including lysosomal health, autophagy, synaptic activity and mitochondrial health [27]. In the following we discuss the evidence linking retromer dysfunction to each of these pathways.

## The retromer-lysosomal degradation axis in Parkinson’s disease

Retromer was initially discovered as a protein complex required for sorting of cargo from the endosome back to the TGN [28]. One classic retromer cargo is the cation-independent mannose 6-phosphate receptor (CI-MPR). This receptor is responsible for the trafficking of newly synthesised hydrolases, such as cathepsin D, from the TGN to the endosomal network and hence ultimately their delivery to the lysosome [29, 30]. Retromer retrieves the unliganded CI-MPR from endosomes back to the TGN, an essential event in maintaining iterative rounds of CI-MPR and hydrolase trafficking [2]. Interestingly, one substrate for cathepsin D mediated lysosomal degradation is alpha-synuclein, a protein prevalent in Lewy bodies, which is another hallmark of PD [31, 32, 33].

Disruption of lysosomal health has been shown to induce accumulation and aggregation of alpha synuclein highlighting this pathway as having an important role in the degradation of alpha synuclein and thereby the pathology of PD [34]. Alpha synuclein has been shown to enter the lysosome via macroautophagy and chaperone-mediated autophagy, both of which are also affected by retromer suppression (see below) [34, 35••, 36•]. One idea that is gaining attention is that retromer dysfunction causes a reduction in the iterative rounds of CI-MPR and hydrolase trafficking leading to a decrease in the efficiency of hydrolase delivery to the lysosome. This would result in lowered lysosomal activity due to a decrease in the hydrolase content in the lysosome potentially leading to the accumulation of non-degraded aggregated proteins (such as alpha synuclein) and de-regulated organelles. Indeed, *in vitro* and *in vivo* suppression of retromer has been shown to affect CI-MPR trafficking leading to abnormal cathepsin D processing and an increase in alpha-synuclein levels [37]. On the flip side, overexpression of VPS35 has been found to be protective against the accumulation and aggregation of alpha-synuclein observed in a synucleinopathy transgenic mouse model as well as in an alpha-synuclein prion-like seeding mouse model [38]. This has been suggested to be due to an increase in the trafficking of relevant hydrolases to the lysosome for efficient protein degradation.

Two VPS35 mutations, p.D620N and p.R524W, have also been shown to impair CI-MPR trafficking and result in dysfunctional cathepsin D processing *in vitro* [22•, 39••, 40••]. In a neuroblastoma cell line this correlates with an increase in alpha-synuclein aggregation, which is consistent with a defect in protein degradation [22^•^]. With the mannose 6 phosphate (M6P) receptors, including the CI-MPR, estimated to be important for the sorting of over 60 different hydrolases it is not difficult to comprehend why dysfunction of retromer-mediated CI-MPR trafficking may lead to a decrease in lysosomal health resulting in impairment of protein degradation [41, 42].

In addition to the M6P receptors two alternative receptors have also been found to have a role in the trafficking of enzymes and proteins to the lysosome: the lysosomal integral membrane protein (LIMP-2) and sortilin [43]. CI-MPR has been shown to associate with LIMP-2 which is responsible for targeting the enzyme β-glucocerebrosidase (β-Gcase) to the lysosome [42]. Mutations within the glucocerebrosidase gene (GBA) cause an enzyme deficiency that leads to the lysosomal storage disorder, Gaucher’s disease. Interestingly, individuals carrying a GBA mutation have a greater risk of developing PD, further suggesting an important role for lysosome de-regulation in PD [44, 45]. Sortilin is linked to various disorders including AD and interestingly, has also been shown to interact with retromer suggesting an additional role for retromer in lysosomal-enzyme trafficking in other neurodegenerative disorders [46].

Another study found that expressing the VPS35(p.D620N) mutation in *vitro* had no effect on CI-MPR trafficking but they did report defects in autophagosome formation and the miss-trafficking of the autophagy protein ATG9A [35^••^] highlighting that other protein degradation pathways may also be affected by retromer. Consistent with this, the lysosome-associated membrane glycoprotein 2a (Lamp2a) is another protein that relies on endosomal retrieval by retromer for its recycling to the TGN. Lamp2a is a receptor for chaperone-mediated autophagy (CMA), which is thought to assist in the lysosomal-mediated degradation of alpha-synuclein [47, 48]. Suppression of retromer or expression of the VPS35(p.D620N) mutation increases the degradation of Lamp2a leading to an increase in alpha-synuclein expression [36^•^]. Indeed, re-expression of Lamp2a into VPS35 deficient dopaminergic neurons prevents alpha-synuclein accumulation, consistent with an important role for CMA and retromer in alpha-synuclein degradation [36^•^].

Overall, the role of retromer in maintaining lysosomal health and the importance that this plays in the efficient degradation of proteins, protein aggregates and de-regulated organelles (via both the endosomal and autophagic pathways) is an emerging theme in the neuroprotective role of retromer (Figure 2).

## The retromer-WASH axis in Parkinson’s disease

To date the primary defect connected with the PD associated VPS35(p.D620N) mutation is a twofold decrease in the affinity of binding between VPS35 and the FAM21 component of the pentameric WASH complex [35••, 39••]. This ancient complex, which in addition to FAM21 contains WASH, SWIP, CCDC53, and strumpellin (a protein that is itself associated with the neurodegenerative movement disorder, hereditary spastic paraplegia), mediates the Arp2/3-dependent nucleation of actin filaments [49, 50, 51]. This is essential in the organisation of functional F-actin subdomains on endosomes, and is required for efficient retromer-dependent and retromer-independent retrieval and recycling of cargo [49]. Interestingly, a mutation in one of a number of WASH accessory proteins, namely DNAJC13 (a.k.a. RME8), has also been linked to familial forms of PD [52, 53], providing evidence for the importance of the retromer-WASH axis in this disease.

Functionally, the suppression of WASH affects the endosomal sorting of the CI-MPR and, as discussed above, perturbed sorting of this receptor is observed with the VPS35(p.D620N) mutation [39••, 40••, 49]. The WASH complex is also involved in autophagasome formation [54, 55] and the trafficking of ATG9A suggesting that the autophagy defects observed in VPS35(p.D620N) cells are due, in part, to a decrease in endosomal recruitment of the WASH complex [35^••^]. The retromer-mediated retrieval and recycling of cargo proteins back to the cell surface also requires the WASH complex [56, 57]. Mislocalisation of the glucose transporter-1 (GLUT-1) in cells expressing VPS35(p.D620N) has been reported which, is consistent with a defect in retromer-mediated endosome to cell surface sorting of this cargo protein [35^••^]. In support of this, the trafficking of the cell surface protein dopamine receptor-1 is affected by the VPS35(p.D620N) mutation in mouse dopaminergic neurons resulting in abnormal dopamine signalling [58^•^]. VPS35(p.D620N) knock-in mice also display impaired dopaminergic neurotransmission in the striatum suggesting a defect in the activity of dopaminergic neurons [59]. In addition, retromer has also been shown to be involved in the trafficking of wntless, a receptor essential for WNT secretion which has also been shown to be involved in dopaminergic neuronal health and PD [60].

AMPA receptor recycling has also been shown to be affected by the VPS35(p.D620N) mutation in rat primary neuronal cultures and in dopaminergic cells derived from patients carrying the p.D620N mutation supporting the idea that changes in the cell surface proteome may play an important role in protecting the cell from degeneration [61^•^]. Studies in yeast have also shown that the VPS35(p.D620N) mutation increases the sensitivity to copper, which has been suggested to be due to the miss-trafficking of copper transporters [62]. Indeed, in human cells endosomal retrieval and recycling of the copper transporter ATP7A is retromer dependent [63]. There is evidence that alterations in copper homeostasis play a role in PD with excess copper leading to neuronal cell death and alpha-synuclein aggregation [64], and so it will be important to consider further the link between retromer and copper homeostasis.

Recently, the VPS26A(p.K297X) mutation has been shown to affect the retromer-WASH mediated endosomal sorting of GLUT-1 through a reduced binding to the cargo adaptor sorting nexin 27 (SNX27) [21•, 65]. SNX27 contains an amino terminal PDZ domain that associates with cargo proteins containing a Type I PDZ domain-binding motif (PDZbm) at their very carboxy-terminus (the PDZbm in GLUT-1 being DSQV) [63, 66••, 67]. Association of SNX27 with VPS26 (and hence the retromer) is via an exposed β-hairpin in the SNX27 PDZ domain that binds to a groove within the arrestin-like structure of VPS26A [68^•^]. Association to VPS26A increases the affinity of SNX27 for its cargo by over 10-fold revealing an allosteric relationship between retromer, SNX27 and cargo recognition [68^•^]. As for cargo recognition, the optimal amino acid sequences of the PDZbm for high affinity binding to SNX27 have been determined [66^••^]. Using these sequence motifs coupled with bioinformatics analysis, it has been determined that the human genome encodes in excess of 400 proteins that are likely cargos for SNX27-retromer mediated endosomal sorting [66^••^], over a hundred of which have been validated [63]. If one combines these data, it is clear that by sorting functional diverse cargos that include ion channels, nutrient transports, and signalling receptors, the SNX27-retromer-WASH axis plays an important role in synaptic plasticity, nutrient uptake and metabolism, development and signal transduction [63, 66••, 69•]. Such a pleiotropic role is entirely consistent with the complex phenotype observed in SNX27 null mice [70] and provides insight into functional effects of reduced SNX27 expression that has been observed in AD, Down’s syndrome and infantile myoclonic epilepsy [71, 72, 73]. Interestingly in the context of cognition, SNX27 regulates the endosomal retrieval and recycling of AMPA and NMDA receptors, with loss of SNX27 leading to effects on excitatory transmission, synaptic function and long term potentiation [70, 71, 74, 75]. Together these studies suggest that disruption of retromer, or its accessory proteins including SNX27 and the WASH complex, can lead to a perturbed steady-state and activity-dependent level of cell surface cargos, leading to perturbed neuronal activity and potentially neuronal viability (Figure 2).

## The retromer-mitochondria axis in Parkinson’s disease

Retromer has been suggested to have a role in mitochondrial health as VPS35 is protective against the neurotoxin MPTP [21^•^]. MPTP converts to MPP+, which by inhibiting complex I of the mitochondrial respiratory chain leads to cell death in the dopaminergic neurons of the SNpc [25, 76, 77]. The mechanism(s) by which retromer is involved in mitochondrial dysfunction however, is currently unclear. VPS35 is argued to localise within subdomains of the mitochondria and has been shown to regulate the trafficking and degradation of MUL1 (MAPL), an E3 ubiquitin ligase enriched in mitochondria-derived vesicles [78]. Mitochondria-derived vesicles are thought to be involved in the transport of cargo from the mitochondria to the peroxisome/lysosome for degradation.

**Figure 3:**
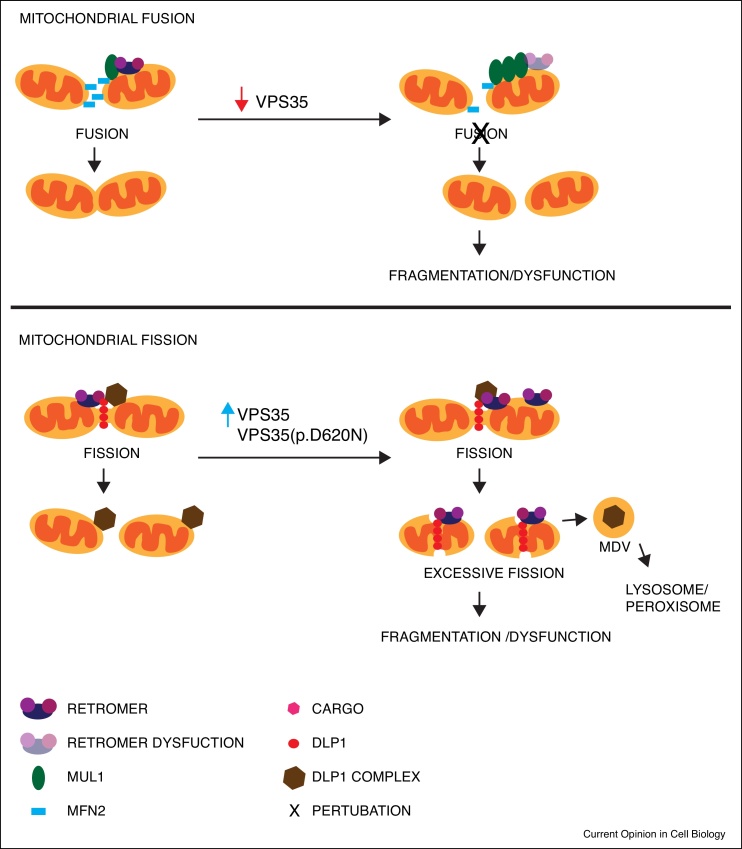
Retromer and mitochondrial fusion and fission. Retromer dysfunction is thought to prevent the degradation of mitochondrial cargo as well as disrupt the balance of mitochondrial fusion/fission leading to neurodegeneration. Retromer has been shown to interact with the mitochondrial E3 ubiquitin protein ligase 1 (MUL1). MUL1 is enriched in mitochondria-derived vesicles and is also involved in mitochondrial fusion through it regulation of the mitochondrial protein mitofusin 2 (MFN2). Retromer suppression causes an increase in MUL1 expression, leading to the increased degradation of MFN2 and mitochondrial fragmentation and dysfunction. In contrast, retromer has also been shown to interact with the mitochondrial fission protein: dynamin-like protein (DLP1). Overexpression of retromer or expression of the VPS35(p.D620N) mutation enhances the interaction of retromer with DLP1. This results in the removal of the DLP-1 complex by mitochondria-derived vesicles for degradation by the lysosome/peroxisome resulting in excessive fission.

Changes in mitochondria fusion and fission have been proposed to play an important role in the pathogenesis of PD. Retromer suppression has been shown to be involved in both fission and fusion but with contrasting effects (Figure 3). One study found that VPS35 could control mitochondrial fusion through its regulation of MUL1 mediated turnover of the GTPase protein mitofusin 2 (MFN2) [79^••^]. MFN2 is essential for mitochondrial fusion, an important event in controlling mitochondrial morphology, function and degradation. Suppression of VPS35 causes an increase in MUL1 expression, which results in the increased degradation of MFN2 leading to mitochondrial fragmentation and dysfunction [79^••^]. Interestingly, suppression of MUL1 not only restores MFN2 levels but also protects against mitochondria dysfunction and remarkably protects against dopaminergic neuronal loss *in vitro* and *in vivo* [79^••^].

In contrast, another study found that retromer suppression causes mitochondria elongation rather than fragmentation which they suggest is due to a decrease in mitochondrial fission rather than an alteration in fusion [80^••^]. Dynamin-like protein (DLP1), a cytosolic mitochondrial fission protein, translocates to the mitochondrial outer membrane and forms a large complex to divide mitochondria. However, the mitochondrial DLP1 complex remains with daughter mitochondria and becomes inhibitory to the subsequent fission. Overexpression of VPS35 or the VPS35(p.D620N) mutation displays an enhanced interaction with the mitochondrial DLP1 complex and promotes the retromer-dependent turnover of fission-inhibitory mitochondrial DLP1 complexes via mitochondria-derived vesicle-dependent trafficking and lysosomal degradation [80^••^]. Recently the retromer-sorting sequence in DLP1 has been identified and a short peptide that interrupts the VPS35-DLP1 interaction has been demonstrated to alleviate VPS35(p.D620N) mutation-induced mitochondrial fragmentation and dysfunction [81^•^].

Interestingly MUL1 has also been shown to SUMOylate and stabilise DLP-1 oligomers at the mitochondria suggesting that retromer may have an important role in the balance of mitochondrial fusion versus fission, which is known to determine mitochondrial health [82]. Moreover, retromer may play an important role in the trafficking of cargo from the mitochondria to the peroxisome/lysosome for degradation, the impairment of which, may also contribute to neurodegeneration (Figure 3) [78]. The precise mechanism by which retromer may be affecting mitochondria however, is currently unclear and more research will be needed to fully understand how retromer dysfunction may be affecting mitochondrial health.

The PD associated genes Pink1 and Parkin have also been shown to be involved in the formation and trafficking of mitochondria-derived vesicles and have been shown to regulate MFN2 and DLP1 ubiquitination and degradation thereby also affecting mitochondrial fusion and fission [58•, 83, 84]. Interestingly, VPS35 and Parkin appear to interact genetically as double mutant heterozygote flies (*vps35^MH20/E42^/+; park^25^/+*) have an increased loss of dopaminergic neurons, increased motor impairment and an increased sensitivity to neurotoxins [85]. The interface between retromer and mitochondrial quality control is an interesting and unexpected observation that in the broader context raises many interesting questions relating to the connectivity and communication between endosomal cargo sorting and mitochondrial function (Figure 2, Figure 3).

## Conclusion

As changes in protein trafficking and degradation are becoming a major focus in the pathology PD, drug targets within the retromer complex and its functional pathways are currently being explored for therapeutic potential [86^••^]. The recent structural characterization of the retromer complex structure will help aid this targeted drug design [87^••^]. Future research will need to further expand our basic understanding of the assembly and function of this complex, while exploring in more precise detail the *in vivo* role of retromer in the maintenance and remodelling of lysosomal health, remodelling of the functional cell surface proteome, and more broadly the role of this protein complex in general proteostasis. Such research is certain to reveal new and exciting insight into neuronal health and disease.

## References and recommended reading

Papers of particular interest, published within the period of review, have been highlighted as:

• of special interest
•• of outstanding interest
